# An Investigation of Diagnostic Accuracy and Confidence Associated with Diagnostic Checklists as Well as Gender Biases in Relation to Mental Disorders

**DOI:** 10.3389/fpsyg.2016.01813

**Published:** 2016-11-22

**Authors:** Jan C. Cwik, Fabienne Papen, Jan-Erik Lemke, Jürgen Margraf

**Affiliations:** Mental Health Research and Treatment Center, Department of Psychology, Ruhr-Universität BochumBochum, Germany

**Keywords:** diagnostic checklist, misdiagnosis, decision-making, gender bias, diagnostic accuracy, diagnostic confidence, therapeutic decisions

## Abstract

This study examines the utility of checklists in attaining more accurate diagnoses in the context of diagnostic decision-making for mental disorders. The study also aimed to replicate results from a meta-analysis indicating that there is no association between patients’ gender and misdiagnoses. To this end, 475 psychotherapists were asked to judge three case vignettes describing patients with Major Depressive Disorder (MDD), Generalized Anxiety Disorder, and Borderline Personality Disorder. Therapists were randomly assigned to experimental conditions in a 2 (diagnostic method: with using diagnostic checklists vs. without using diagnostic checklists) × 2 (gender: male vs. female case vignettes) between-subjects design. Multinomial logistic and linear regression analyses were used to examine the association between the usage of diagnostic checklists as well as patients’ gender and diagnostic decisions. The results showed that when checklists were used, fewer incorrect co-morbid diagnoses were made, but clinicians were less likely to diagnose MDD even when the criteria were met. Additionally, checklists improved therapists’ confidence with diagnostic decisions, but were not associated with estimations of patients’ characteristics. As expected, there were no significant associations between gender and diagnostic decisions.

## Introduction

A comprehensive and systematic diagnostic process based on objective criteria is of particular importance for the treatment of mental disorders (Ramirez Basco et al., 2000; Ehlert, 2007). Nevertheless, several studies have shown high rates of misdiagnoses in daily practice (Kales et al., 2005; Bruchmüller and Meyer, 2009; Wolkenstein et al., 2011). Though patient factors, such as gender, are frequently assumed by clinicians to be a cause of misdiagnosis, in reality, among other factors, clinicians’ diagnostic approach is a more prominent cause of misdiagnosis (Zimmerman and Mattia, 1999; Wolkenstein et al., 2011; Cwik and Teismann, 2016). The diagnostic process may involve subjective descriptions of the patient or may influence or be influenced by therapists’ expectations (Langer and Abelson, 1974; Rosenhan and Seligman, 1989; Margraf and Schneider, 2009). Clinicians may also tend toward looser interpretation and use of the diagnostic criteria of classification systems and might resort to other resources instead, such as their professional experiences or personal assumptions (Morey and Ochoa, 1989; Bruchmüller and Meyer, 2009; Meyer and Meyer, 2009; Wolkenstein et al., 2011; Bruchmüller et al., 2012; Garb, 2013; Cwik and Teismann, 2016). As a result, in single diagnoses, clinicians tend to be more likely to make false-positive diagnoses over time, assigning a disorder label though not all required diagnostic criteria are fulfilled (Bruchmüller et al., 2011). Regarding comorbidity, the opposite tends to happen: the most salient diagnosis becomes diagnosed and comorbid disorders are missed (Garb, 1998).

Structured diagnostic interviews are recommended in clinical practice to safeguard against misdiagnosis due to clinician bias (Schneider and Döpfner, 2004; Joiner et al., 2005; Silverman and Ollendick, 2005; Ehlert, 2007). Although interviews do help to reduce bias and misdiagnosis (Margraf and Schneider, 2009), clinicians rarely use such instruments in daily practice (In-Albon et al., 2008; Suppiger et al., 2008, 2009; Bruchmüller et al., 2011). Therapists provided varying rationales for not using structured interviews: their clinical judgment is more useful (37%) and the length of the interviews (34%).

Diagnostic checklists provide an alternative to interviews and represent a compromise between clinicians’ preference for less structured discussion and the inclusion of specific diagnostic criteria in the diagnostic process, enabling an evaluation process of higher reliability and validity than the status quo (Hiller et al., 1993). Diagnostic checklists are beneficial in that they can assess diagnostic criteria without interfering with the clinician’s rapport or interview approach. Thus, open clinical judgment (OCJ) is still possible, but in combination with the assurance that all criteria of a potential disorder are considered systematically and that non-conformity with the criteria of a diagnosis is consciously noted. There is evidence that diagnostic checklists can help to attain reliable diagnoses and to increase diagnostic accuracy (Biederman et al., 1993; Bronisch and Mombour, 1994; Aebi et al., 2010; Mokros et al., 2013; Vaughn and Hoza, 2013).

However, accuracy of given diagnoses is only one essential component for a well-considered psychotherapy. Other important aspects are treatment planning, confidence with given diagnoses, and the estimation of therapy relevant patient characteristics (Brownstein, 2003; Schmidt et al., 2005; Arvilommi et al., 2007; Bruchmüller and Meyer, 2009; Meyer and Meyer, 2009). However, Witteman and Kunst (1997) showed that professionals tend to insist on their first clinical opinion and then search for confirming information. Assuming that different therapists have different underlying confirmation biases, therapeutic recommendations and estimations of patients’ characteristics may vary when therapists do not use diagnostic tools to ensure a standardized consideration of all relevant aspects. In line with this, study results showed that neglecting diagnostic relevant information leads to inappropriate treatment planning (Drake et al., 1993; North et al., 1997). Furthermore, therapists suggest that the consideration of DSM criteria is not important for treatment planning (Zimmerman et al., 1993) and that their clinical judgment is more useful than diagnostic tools (Bruchmüller et al., 2011). More recently, Zimmerman (2016) pointed out in his systematic review that accurate diagnostics may have an impact on treatment recommendations.

Based on the assumption that the diagnostic process has an effect on treatment (Ramirez Basco et al., 2000; Ehlert, 2007) and that an effective diagnostic process could improve treatment planning (Haynes and Williams, 2003; Groenier et al., 2008), one could assume that diagnostic checklists – as effective diagnostic tools – will promote accurate diagnostics and adequate treatment recommendations and estimation of patient characteristics compared to OCJ. Consequently, we hypothesized that the usage of diagnostic checklists influences diagnosticians’ confidence with given diagnoses and other therapeutic aspects, such as taking patient characteristics or diagnosticians’ appraisal of the subsequent psychotherapeutic process into consideration.

In addition to diagnostic methods, gender biases and their association with misdiagnoses of mental disorders became of scientific interest in the 1970s (Broverman et al., 1970). Currently and historically, several diagnoses tend to be more prevalent in one gender, with a bias toward more prevalence in general in women (American Psychological Association [APA], 1975, 1978). Mental disorders with increased prevalence in one gender are of particular interest to psychiatrists and psychotherapists. Major Depressive Disorder (MDD), Generalized Anxiety Disorder (GAD), and Borderline Personality Disorder (BPD) have shown a greater prevalence in women (Angst et al., 2002; Wittchen and Jacobi, 2005; Morschitzky, 2009; Seedat et al., 2009), with some potential overdiagnosis.

Widiger and Spitzer (1991) postulated that gender-biased mis- or over-diagnosis can occur on two levels: in relation to the application of the diagnostic criteria and/or related to the diagnostic criteria themselves. In line with this, some evidence indicates that a diagnostic category that is, in clinician’s opinion, linked to a particular gender, is more likely to be given when the symptomatic behavior of the patient is concordant with traditional gender stereotypes (Sprock, 1996; Crosby and Sprock, 2004; Flanagan and Blashfield, 2005). Clinicians tend to pathologize symptoms when there is a large gap between symptoms and traditional gender characteristics of a patient (Sprock, 1996; Möller-Leimkühler, 2005). For instance, Möller-Leimkühler (2005) reports that emotional expressions in a depressive man or aggressive behavior in a depressive woman are not in line with traditional gender characteristics and are associated with misdiagnosis of MDD. According to these findings, Ford and Widiger (1989) illustrated the effects of gender biases in a study on personality disorders. They showed that even if women fulfilled the diagnostic criteria for Antisocial Personality Disorder, clinicians more frequently diagnosed Histrionic Personality Disorder, despite non-fulfillment of the diagnostic criteria.

Assuming that therapists are aware of the connection between gender and prevalence, one could expect consistent gender biases in diagnostic decisions. However, evidence of the influence of gender stereotypes on diagnostic decisions is mixed (Abramowitz et al., 1976; Gomes and Abramowitz, 1976; Teri, 1982; Heatherington et al., 1986; Hansen and Reekie, 1990). For example, therapists made significantly more MDD diagnoses when the described person in a case vignette was a woman in comparison to a man (Wrobel, 1993; Bertakis et al., 2001; Lewis et al., 2006). However, other studies reported no effect of patient’s gender in MDD (Hansen and Reekie, 1990; López et al., 1993; Case et al., 1999; Kales et al., 2005). More generally, Garb (1997) found that women are no more likely to be given a mental health diagnosis than men. Additionally, a recent meta-analysis (Cwik et al., under review) of ours showed that patient’s gender is not a causal factor for misdiagnoses of mental disorders when examining results across 22 studies.

The aim of the present study was to investigate two main questions. First, we wanted to investigate diagnostic accuracy when using diagnostic checklists for mental disorders as compared with OCJ. Additionally, we were interested in finding out to what extent the usage of checklists increases diagnosticians’ confidence with given diagnoses. We hypothesized that clinicians’ diagnostic accuracy and confidence would be higher when using checklists.

Secondly, this study aimed to investigate the relationship between misdiagnoses and patients’ gender. Based on the results of our meta-analysis, one could assume that a diagnostic gender bias is absent when it is investigated in mental disorders in general, but present in specific mental disorders that are more frequent in females (e.g., MDD, GAD, BPD). Even in these disorders, we expected that patients’ gender is not a cause of misdiagnosis or of different estimations of diagnosticians’ confidence with given diagnoses.

Finally, we conducted an exploratory analysis of how the usage of checklists and patients’ gender is associated with the following diagnostic aspects: clinicians’ estimation of the severity of diagnoses, patients’ motivation for treatment, expected number of treatment sessions until a significant improvement of symptoms, and clinicians’ recommendation for therapeutic interventions.

## Materials and Methods

The Ethics Committee of the Ruhr-Universität Bochum approved the study. All participants gave their informed consent before they were able to start with the questionnaire.

### Participants and Procedure

An invitation to the study was send out to mailing addresses of psychotherapists that were available by using the search functions of the Chamber of Psychotherapists in all federal states of Germany and of the “Deutsche Psychotherapeutenvereinigung” (German Association of Psychotherapists). In this email, participants received background information about the study and received a link to the online survey. The online survey was completed anonymously and participants could only proceed to the next questionnaire once all questions had been answered. A response of 834 invitees was achieved. Participants were randomly allocated to conditions. The exclusion rate was 359, due to incompletion of 357 (42.81%) diagnostic surveys and implausible information of two surveys, thus an effective response rate of *n* = 475 (56.95%) was achieved [checklist (CL): *n* = 245 vs. OCJ: *n* = 230; female vignettes: *n* = 241 vs. male vignettes: *n* = 234]. The exclusion of the incomplete surveys ensured that there was no missing data in the remaining 475 surveys. The data collection took place between April and August 2011.

Participants’ mean age was 46.67 years (*SD* = 11.02), and 326 participants (68.8%) were women. The sample comprised 79.2% clinicians who had completed cognitive behavioral psychotherapy training, 22.5% who had completed training in analytic psychotherapy, and 24.6% who had completed another type of psychotherapeutic training (including gestalt, client-centered, systemic therapists). Of all psychotherapists, 75.4% had completed a single therapeutic training (e.g., only a training in CBT), 22.9% had completed two trainings (e.g., trainings in CBT and gestalt therapy), and 1.7% had completed three (e.g., trainings in CBT, gestalt therapy, and psychoanalysis) or more trainings. Overall, 82.9% of the participants worked in their own practice, with a mean of 16 years (*SD* = 10.13) of professional experience. When asked to rate their clinical experience in using the ICD-10 (World Health Organization [WHO], 1992) and the DSM-IV-TR (American Psychiatric Association [APA], 2000) (1 = “*no experience*” to 5 = “*very experienced”)*, the participants rated their level of experience with ICD-10 higher (*M* = 4.37, *SD* = 0.71) than their experience with DSM-IV-TR (*M* = 2.53, *SD* = 1.09); there was a significant difference between experiences with both classification systems [*t*(474) = -32.35, *p* < 0.001].

### Materials

#### Case Vignettes

Each participant received an online survey with a cover letter and a link to the survey including three case vignettes (available as Supplementary Material). The case vignettes were constructed on the basis of DSM-IV-TR and were extended to include essential criteria for ICD-10, so that the underlying disorders could be unambiguously diagnosed according to both classification systems.

The first case vignette described a middle-aged patient with a severe episode of MDD. The vignette contained all information necessary to clearly diagnose a MDD episode without psychotic symptoms, according to DSM-IV-TR and ICD-10, except the criterion for suicidal behavior. The second vignette described a patient fulfilling criteria for GAD, based on a DSM-III case description (Spitzer et al., 1991). For this case vignette, we shortened the original description attuned to German culture and to DSM-IV-TR criteria. To ensure that participating therapists who usually use ICD-10 diagnostic criteria in their daily routine would be able to diagnose GAD, we added all required symptoms of ICD-10. The last vignette described a patient fulfilling all general criteria for a personality disorder according to DSM-IV-TR as well as seven required criteria of a BDP. It was based on a case description of Zaudig et al. (2000).

To validate the diagnostic criteria of the underlying disorders, seven licensed psychotherapists reviewed all vignettes. All psychotherapists diagnosed the correct disorder without an additional (comorbid) diagnosis in each vignette.

#### Diagnostic Questionnaire

All participants received the same diagnostic questionnaire. In this questionnaire, therapists were asked to diagnose the case vignettes. They were able to choose up to three options. One option of which was “*no disorder present.*” The other options were 12 listed diagnoses. In addition, therapists of both groups were asked how reliable they rated the selected diagnosis (0 = “*insufficient information*” to 100 = “*very reliable*”).

Furthermore, therapists were asked to rate the assumed extent of mental, social and job-related impairment caused by the described symptoms (0 = “*mentally healthy*” to 100 = “*mentally ill*”), patients’ motivation for a therapeutic treatment (0 = “*not at all motivated*” to 100 = “*highly motivated”*), and severity of the described disorder (1 = “*no mental disorder*” to 8 = “*severe mental disorder*”), and were asked to estimate how many treatment sessions would be needed until significant improvement could be expected. Finally, therapists were asked which therapeutic orientation for treatment they would advise.

#### Diagnostic Checklists

Checklists were only presented to participants in the checklist condition. Initially, the checklists were not visible to participants. First participants had to choose and click on a diagnosis that determined the checklist they would be presented. For instance, if a participant decided for MDD and clicked on MDD diagnosis, the corresponding MDD checklist opened. Then the participant was able to verify the given diagnosis or to falsify it, switch to another diagnosis and receive the corresponding checklist (e.g., choosing Dysthymia diagnosis and receiving the checklist for Dysthymia).

The checklists listed particular keywords extracted from DSM-IV-TR criteria for each diagnosis. Subsequent to each symptom, participants had to decide whether the symptom was described or not. Finally, at the end of the checklist, participants had to decide whether the criteria were fulfilled.

### Statistical Analysis

Although participants were initially randomized, due to exclusion of incomplete 359 surveys, several between-group significant differences in demographic variables were observed. For the diagnostic procedure condition, significant between-group differences were observed several demographic variables. Significant group differences were observed in: age [*t*(473) = -6.80; *p* < 0.001], professional experience [*t*(473) = -5.60; *p* < 0.001], occupational setting [*t*(473) = 4.17; *p* < 0.001], and therapeutic direction (behavior therapy) [*t*(473) = -1.03; *p* = 0.034]. For the gender condition, significant between-group differences were observed in age ([*t*(473) = -4.86; *p* < 0.001], professional experience [*t*(473) = -5.60; *p* < 0.001], occupational setting [*t*(473) = 4.31; *p* < 0.001], and clinical practice in usage of ICD-10 [*t*(473) = -2.65; *p* = 0.008]). For all other demographic variables, no significant differences between groups were observed. These significant variables were used for the calculation of propensity scores (PS) for each condition: a PS for each participant for gender condition and another PS for diagnostic procedure condition. The PS were calculated using logistic regression with the respective conditions as dependent variables and the demographic variables showing significant differences between groups as independent variables (Rosenbaum and Rubin, 1983; D’Agostino, 1998; Bartak et al., 2009; Weinberger et al., 2009; Austin, 2011).

Next, multinomial logistic regression analyses were performed for each case vignette, with each of the two conditions (diagnostic procedure and patients’ gender) as independent variables and diagnostic decisions and treatment recommendations as dependent variables. Within each condition, the corresponding PS for that condition were used for weighting each participant’s contribution, to control for significant group differences.

Additionally, multiple linear regression analyses were used to analyze the nature of correctly diagnosed cases within each case vignette after weighting for differences in demographic variables. The respective conditions (diagnostic procedure and patient’s gender) were used as independent variables. Therapists’ confidence of diagnosis, patients’ motivation for treatment, severity of diagnosis and the expected number of treatment sessions were included as dependent variables. Furthermore, model fit and relative strength of associations between conditions and diagnostic decisions were assessed using Nagelkerke’s *R*^2^ and Cohen’s *f*^2^.

All results were Bonferroni-corrected for multiple comparisons. Data analysis was conducted using SPSS version 22.0 for Mac (IBM Corporation, 2013).

## Results

### Diagnostic Decisions

#### Checklists and Diagnostic Decisions

As can be seen in **Table 1**, results from the multinomial logistic regression analysis revealed that the usage of OCJ was significantly associated with making more false comorbid diagnoses in patients with MDD (*p* < 0.005), GAD (*p* < 0.001), and BPD (*p* < 0.001), compared to making the correct diagnosis and to therapists using checklists. Furthermore, the usage of OCJ was also significantly associated with making false diagnoses in patients with GAD (*p* < 0.001) and patients with BPD (*p* = 0.002), compared to making the correct diagnosis and to therapists using checklists.

**Table 1:** Results of three multinomial logistic regression analyses associating usage of checklists and patients’ gender with diagnostic decisions (reference category = correct diagnostic decision).

	Comparisons				Model fit index			
Vignette	Condition	comorbid vs. correct	false vs. correct	no vs. correct	
MDD		*OR*^a^ [95% *CI*]	*OR*^a^ [95% *CI*]	*OR*^a^ [95% *CI*]	X^2^(*df)*	*p*	*N*	Nagelkerke *R*^2^
	Procedure	**2.62 [1.34 – 5.10]^∗^**	1.81 [0.88 – 3.75]	**0.05 [0.01 – 0.21]^∗∗^**	82.73 (12)	<0.001	475	0.19
	Gender	1.89 [1.01 – 3.56]	0.90 [0.46 – 1.78]	0.88 [0.42 – 1.83]				
GAD		*OR*^a^ [95% *CI*]	*OR*^a^ [95% *CI*]	*OR*^a^ [95% *CI*]	X^2^(*df)*	*p*	*N*	Nagelkerke *R*^2^
	Procedure	**5.34 [2.68 – 10.64]^∗∗^**	**5.06 [2.61 – 9.83]^∗∗^**	0.61 [0.25 – 1.42]	96.39 (12)	<0.001	475	0.21
	Gender	1.12 [0.62 – 2.04]	0.70 [0.39 – 1.23]	1.17 [0.51 – 2.67]				
BPD		*OR*^a^ [95% *CI*]	*OR*^a^ [95% *CI*]	*OR*^a^ [95% *CI*]	X^2^(*df)*	*p*	*N*	Nagelkerke *R*^2^
	Procedure	**4.81 [2.83 – 8.17]^∗∗^**	**5.01 [1.82 – 13.80]^∗∗^**	1.33 [0.73 – 2.45]	58.86 (12)	<0.001	475	0.13
	Gender	1.42 [0.87 – 2.31]	0.56 [0.23 – 1.35]	0.98 [0.53 – 1.78]				

**OR*, odds ratio; 95% *CI*, 95% confidence interval; MDD, Major Depressive Disorder; GAD, Generalized Anxiety Disorder; BPD, Borderline Personality Disorder; correct, correct diagnosis given; comorbid, correct diagnosis given with a false comorbid diagnosis; false, only false diagnosis/diagnoses given; no, no diagnosis/diagnoses given. Significant results are presented in bold letters and were all Bonferroni corrected: ^∗^*p* < 0.016; ^∗∗^*p* < 0.003. ^a^Adjusted for significant differences demographic data by using propensity scores.*

Contrary to our hypothesis that clinicians’ diagnostic accuracy would be higher when using checklists, the usage of checklists was significantly associated with therapist’s decision to refrain from making a diagnosis in the MDD case, compared to making correct diagnostic decisions and therapists using OCJ (*p* < 0.001). This means that there were more false-negative diagnoses when using checklists compared to using OCJ.

#### Patients’ Gender and Diagnostic Decisions

To examine the association between patients’ gender and diagnostic decisions, all three case vignettes were given to participants either in a male or in a female version. As indicated in **Table 1**, there was no significant association between patients’ gender and giving false comorbid diagnoses, false diagnoses or no diagnoses in patients with MDD, GAD, or BPD.

### Treatment Recommendations

#### Checklists and Therapists’ Treatment Recommendations

With respect to treatment recommendations, there was no significant association between the diagnostic procedure and recommendations in the MDD and the GAD cases. Pertaining to the treatment recommendations in the BPD case, therapists who used checklists recommended significantly more frequently dialectic-behavioral therapy (DBT) as preferable therapy (*p* = 0.007) than psychotherapists in the OCJ condition (**Table 2**).

**Table 2:** Results of three multinomial logistic regression analyses associating usage of checklists and patients’ gender with treatment recommendations (reference category = CBT/DBT).

	Comparisons				Model fit index			
Vignette	Condition	CT vs. CBT/DBT	AT vs. CBT/DBT	OTH vs. CBT/DBT				
MDD		*OR*^a^ [95% *CI*]	*OR*^a^ [95% *CI*]	*OR*^a^ [95% *CI*]	X^2^(*df)*	*p*	*N*	Nagelkerke *R*^2^
	Procedure	0.87 [0.33 – 2.29]	1.26 [0.72 – 2.19]	0.96 [0.58 – 1.61]	66.66 (12)	<0.001	475	0.15
	Gender	0.83 [0.32 – 2.19]	1.09 [0.63 – 1.89]	1.16 [0.69 – 1.93]				
GAD		*OR*^a^ [95% *CI*]	*OR*^a^ [95% *CI*]	*OR*^a^ [95% *CI*]	X^2^(*df)*	*p*	*N*	Nagelkerke *R*^2^
	Procedure	0.56 [0.29 – 1.11]	1.06 [0.58 – 1.92]	1.21 [0.72 – 2.02]	56.84 (12)	<0.001	475	0.13
	Gender	0.80 [0.41 – 1.56]	1.49 [0.81 – 2.72]	1.18 [0.71 – 1.97]				
BPD		*OR*^a^ [95% *CI*]	*OR*^a^ [95% *CI*]	*OR*^a^ [95% *CI*]	X^2^(*df)*	*p*	*N*	Nagelkerke *R*^2^
	Procedure	1.11 [0.58 – 2.12]	1.81 [0.70 – 3.55]	**2.54 [1.38 – 4.70]^∗∗^**	55.58 (12)	<0.001	475	0.13
	Gender	0.85 [0.44 – 1.63]	1.37 [0.70 – 2.67]	0.83 [0.47 – 1.48]				

**OR*, odds ratio; 95% *CI*, 95% confidence interval; MDD, Major Depressive Disorder; GAD, Generalized Anxiety Disorder; BPD, Borderline Personality Disorder; CBT, cognitive-behavioral therapies; CT, cognitive therapies; AT, analytic therapies; OTH, other therapies; DBT, dialectic-behavioral therapy. Significant results are presented in bold letters and were all Bonferroni corrected: ^∗∗^*p* < 0.003. ^a^Adjusted for significant differences demographic data by using propensity scores.*

#### Patients’ Gender and Therapists’ Treatment Recommendations

With respect to treatment recommendations, there was no significant association between the patients’ gender and treatment recommendations (**Table 2**).

### Confidence with Given Diagnoses and Estimations of Patients’ Characteristics

Multiple linear regression analyses were conducted, using both conditions as independent variables and the appraisal of patients’ motivation for treatment, severity of given diagnoses, confidence in diagnoses, and psychotherapists’ estimation regarding the expected number of treatment sessions until the patient experiences significant improvement as dependent variables. Additionally, corresponding PS were included in the regression models to control for significant differences in demographic variables.

#### Checklists and Therapists’ Confidence and Estimations of Patients’ Characteristics

As can be seen in **Table 3**, there was a significant association between the usage of checklists and the therapist’s confidence in the diagnosis in all case vignettes (MDD: β = -0.161; *p* < 0.001; GAD: β = -0.206; *p* < 0.001; BPD: β = -0.205; *p* < 0.001).

**Table 3:** Results of the multiple linear regression models associating usage of checklists and patients’ gender with confidence of diagnoses, motivation for treatment, severity of diagnoses and number of expected treatment sessions.

				Model fit index				Effect size
MDD vignette	Condition	β^a^	*p*	*R^2^*	Adjusted *R^2^*	*F(df)*	*p*	*f^2^*
Confidence of diagnosis (0–100)	Procedure	**-0.161**	**0.001^∗^**	0.036	0.028	4.44 (4, 470)	0.002	0.037
	Gender	-0.006	0.905					
Motivation for treatment (0–100)	Procedure	0.101	0.035	0.040	0.032	4.96 (4, 470)	0.001	0.042
	Gender	**-0.142**	**0.003^∗^**					
Severity of diagnosis (1–8)	Procedure	-0.113	0.017	0.056	0.048	7.00 (4, 470)	<0.001	0.059
	Gender	0.046	0.333					
Number of sessions	Procedure	-0.068	0.154	0.034	0.026	4.10 (4, 470)	0.003	0.035
	Gender	0.072	0.131					
**GAD vignette**								
Confidence of diagnosis (0–100)	Procedure	**-0.206**	**<0.001^∗^**	0.041	0.033	5.06 (4, 470)	0.001	0.043
	Gender	0.021	0.651					
Motivation for treatment (0–100)	Procedure	0.029	0.553	0.008	-0.001	0.91 (4, 470)	0.456	0.008
	Gender	-0.051	0.286					
Severity of diagnosis (1–8)	Procedure	-0.055	0.240	0.074	0.067	9.46 (4, 470)	<0.001	0.080
	Gender	0.030	0.525					
Number of sessions	Procedure	0.012	0.802	0.019	0.011	2.32 (4, 470)	0.056	0.019
	Gender	0.027	0.569					
**BPD vignette**								
Confidence of diagnosis (0–100)	Procedure	**-0.205**	**<0.001^∗^**	0.049	0.041	6.08 (4, 470)	<0.001	0.052
	Gender	-0.073	0.124					
Motivation for treatment (0–100)	Procedure	-0.038	0.422	0.029	0.021	3.52 (4, 470)	0.008	0.030
	Gender	**-0.159**	**0.001^∗^**					
Severity of diagnosis (1–8)	Procedure	-0.030	0.537	0.020	0.012	2.41 (4, 470)	0.048	0.020
	Gender	0.085	0.076					
Number of sessions	Procedure	-0.056	0.245	0.031	0.023	3.80 (4, 470)	0.005	0.032
	Gender	0.107	0.025					

*MDD, Major Depressive Disorder; GAD, Generalized Anxiety Disorder; BPD, Borderline Personality Disorder. Significant results are presented in bold letters and were all Bonferroni corrected: ^∗^*p* < 0.004. ^a^Adjusted for significant differences in demographic data by using propensity scores.*

With regard to the estimation of disorders’ severity ratings and patients’ motivation for treatment as well as the estimation of expected treatment sessions, no significant associations were found.

#### Patients’ Gender and Therapists’ Confidence and Estimations of Patients’ Characteristics

As **Table 3** illustrates, there was a significant association between men with MDD and men with BPD and an appraisal of lower motivation for treatment (MDD: β = -0.142, *p* = 0.003; BPD: β = -0.159, *p* = 0.001), but not in case of men with GAD (β = -0.051, *p* = 0.286). With respect to the confidence with diagnostic decisions, the estimation of the severity of the diagnoses and the number of expected treatment sessions, there were no significant associations observed with patients’ gender in any of the three cases.

## Discussion

The present study examined the diagnostic accuracy of psychotherapists with the use of checklists in the diagnostic process and investigated diagnostic accuracy as related to patient gender. We hypothesized that the usage of checklists would result in significantly higher diagnostic accuracy. After controlling for group differences in several therapist demographic variables and therapist training/experience, this hypothesis was mostly supported by the data with one exception: for the MDD case vignette, when using diagnostic checklists therapists made significantly more false-negative diagnoses compared to when using OCJ.

With regard to the association between the use of checklists and misdiagnoses, psychotherapists who used OCJ were more likely to diagnose an additional comorbid disorder in all of the three disorder cases. In the literature on misdiagnoses, there is evidence that therapists base diagnoses more on comparison to prototypes than on the meeting of diagnostic criteria (Blashfield and Herkov, 1996; Garb, 1996; Westen and Shedler, 2000; Crosby and Sprock, 2004). This could explain the difference between the diagnostic decisions of therapists who used checklists and those that did not.

Furthermore, it seems that therapists rely on various heuristics while making a diagnosis, based on their professional experience and, as prior studies also showed, their appraisal of symptoms and diagnoses is not often evident (Morey and Ochoa, 1989; Blashfield and Herkov, 1996; Crosby and Sprock, 2004). In their theory of heuristics and biases, Tversky and Kahneman (1974) point out that even experts are prone to errors of judgment. Concerning the prevalence and the overdiagnosing of Mood Disorders in this study, it seems that psychotherapists seem to be prone to a representativeness bias (Tversky and Kahneman, 1974; Blumenthal-Barby and Krieger, 2015) with respect to their knowledge of the prevalence and regarding the symptoms of mood and hyperarousal reported in the vignettes. It also seems that the last mentioned aspect triggers a focus on particular symptom complexes that provoke a neglect effect with respect to other relevant symptoms. Furthermore, it seems that the representativeness bias draws diagnosticians’ attention to particular symptom complexes, while other relevant symptoms are neglected. Regarding representativeness bias, it could be surmised that the less frequent the diagnosis, and thus, the less familiar therapists are with the diagnosis, the more they will benefit from using checklists to attain correct and to avoid false diagnoses.

Contrary to our expectations, psychotherapists were more likely to refrain from making a diagnosis in the MDD case vignette when they used checklists. The results of a study by Garb (2007) showed that the usage of computer-administered interviews and checklists was associated with more false-positive diagnoses. Thus, Garb recommends a combination of such a diagnostic instrument with clinical judgment.

More information was collected using the computer-administered interviews and checklists than traditional clinical interviews. Admittedly, in the study of Garb (2007), more symptoms were revealed and thus, it is not surprising that more diagnoses were made. Contrary, in the present study checklists were used to help clinicians making diagnoses from vignettes and thus, this study is involved more with data integration than data collection. Furthermore, although psychotherapists were given checklist information, they were not given the diagnostic criteria for MDD. As Garb (1996) illustrated, if clinicians make diagnoses by comparing clients to prototypes, they may not know the diagnostic criteria for MDD and may have continued to compare the MDD case vignette to their prototype for MDD. Then, if the checklist contained information psychotherapists do not usually collect, that information may not be part of their prototype, and thus the vignette may seem more dissimilar to their prototype resulting in fewer diagnoses of MDD. Thus, one could assume that giving clinicians checklist information does not mean the representativeness heuristic is no longer descriptive. However, it is possible that they were unable to clarify uncertainties with respect to single symptoms. In the case of doubt, they might have become more conservative in their judgment and decided to refrain from making a diagnosis. To deal with this problem, it could be helpful to provide additional lists with exemplary descriptions. However, Margraf and Schneider (2009) pointed out, while referring to the study of Wittchen and Unland (1991), that checklists do not protect against confirmation bias in the diagnostic process. Therefore, the reliability and validity of diagnoses based on the usage of checklists depends on the clinician’s training as well as on the homogeneity of the patients.

Concerning the recommendation of DBT in the BPD case, therapists who used checklists recommended DBT two and a half times as often as therapists who made a diagnosis based on OCJ. It is possible that checklists help therapists to maintain an overview of the full extent of the disorder, whereas therapists relying on OCJ may neglect problem areas or misjudge symptoms. Thus, therapists using checklists tend to consider a broad range of problems, especially cognitive and behavioral deficits, while simultaneously considering BPD specific dysfunctional behaviors like self-injury. Therefore, therapists using checklists were prepared to recommend an adequate therapeutic intervention (American Psychiatric Association [APA], 2005).

Regarding the hypothesis that the use of checklists is significantly associated with higher confidence with diagnostic decisions, the results of the study confirm our hypothesis: Therapists who used checklists reported significantly higher confidence with their diagnostic decisions in all three case vignettes compared to psychotherapists who did not use checklists. The results indicate that even a low-threshold diagnostic instrument such as a checklist is associated with increased diagnostician certainty when compared to OCJ, which is in line with prior results (Vicente et al., 2007). Considering the fact that psychotherapists made significantly more false-positive diagnoses, it is questionable whether higher confidence with diagnostic decisions related to the use of checklists is desirable.

We investigated the association of patients’ gender with the accuracy of diagnostic decisions. Based on the results of our meta-analysis, we expected that there would no significant association between patients’ gender and diagnostic decisions in all three case vignettes. In line with our expectations, we generally found no significant association between patients’ gender and diagnostic decisions of psychotherapists or treatment recommendations. However, an association was found between therapists’ estimations of motivation for treatment in male patients with MDD and BPD. Admittedly, the choice of disorders that were described in the case vignettes could explain this result. Alternatively, in the 1970s and 1980s, diagnosticians may also have become more aware of gender-related biases and thus tried to counteract them.

However, Widiger and Spitzer (1991) postulated that biases due to patients’ gender can occur on two levels: on application of the diagnostic criteria and on the diagnostic criteria themselves. Accordingly, clinicians are more likely to make gender-linked diagnoses if patients’ symptomatic behaviors correspond to gender stereotypes (Sprock, 1996; Crosby and Sprock, 2004; Flanagan and Blashfield, 2005). Likewise, experts tend to pathologize when there is a great gap between patients’ symptoms and traditional gender characteristics (Sprock, 1996; Möller-Leimkühler, 2005). A number of stereotypes are reported, e.g., in relation to differences in symptom expression between men and women (Vredenburg et al., 1986). The case vignettes used in our study did not revert to different these aspects and thus, generalization of these results may be more limited. Future studies should therefore consider these serotype-related misdiagnoses in their case descriptions.

Finally, it should be mentioned that diagnostic criteria vary between ICD and DSM and that there is no gold standard for making “correct” diagnoses. Thus, simply showing that the usage of checklists can improve diagnosis by using the very criteria that have been used to make the classification of the disorder, is in itself not sufficient to improve the diagnostic process or improve decisions on adequate therapy. As recommended by Garb (2007) a combination of such diagnostic instruments with clinical judgment of a therapists is therefore highly recommended. Accordingly, diagnostic instruments – like diagnostic checklists or interviews – should be seen as helpful diagnostic tools, but they should be clinician administered.

### Limitations

A limitation of this study may be the use of case vignettes. Thus, therapists were not able to request additional information during the diagnostic process and might have been limited in their decisions. Future studies should analyze the diagnostic process in detail and inquire as to which information therapists would additionally need for their diagnostic decisions. Studies may also examine why therapists decided not to make a diagnosis (e.g., which criterion was not fulfilled). This could clarify why therapists using checklists more often refrained from making a diagnosis, although a mental disorder was evident. Furthermore, the checklists used were created specifically for this study. Thus, the results regarding the effects of checklists may be limited to these specific checklists. Finally, future studies should include a greater selection of possible diagnoses and investigate comorbid diagnoses to illustrate more ecologically valid results.

## Conclusion

In sum, the present study supports research indicating that diagnostic checklists can improve the accuracy of mental disorder diagnoses, and could be a useful tool to avoid false-positive diagnostic decisions. Nevertheless, the results of our study revealed that the use of checklists could also lead to more false-negative diagnoses when compared with OCJ. Furthermore, the use of checklists provides a higher level of confidence in diagnostic decisions compared with OCJ and is also associated with more correct treatment recommendations.

With regard to the investigation of an association between patients’ gender and misdiagnoses, the results of this study revealed no gender bias and no association in relation to most diagnostic decisions.

## Ethic Standards

The study was approved by the Ethics Committee of the Faculty of Psychology at the Ruhr-Universität Bochum.

## Author’S Disclosure

All authors read and approved the final manuscript and agreed to authorship. All authors state their compliance with the Code of Ethics of the World Medical Association (Declaration of Helsinki). They also agree to the ethical standards of the Faculty of Psychology’s Ethical Commission of the Ruhr-Universität Bochum.

## Author Contributions

Conceived the study: JM and JC. Accessed the data: FP and J-EL. Data analysis: JC. Written text: JC and JM. Interpretation of findings and manuscript revision: JC, FP, J-EL, and JM.

## Conflict of Interest Statement

The authors declare that the research was conducted in the absence of any commercial or financial relationships that could be construed as a potential conflict of interest.

## References

[B1] Abramowitz S. I. Roback H. B. Schwartz J. M. Yasuna A. Abramowitz C. V. Gomes B. (1976). Sex bias in psychotherapy: a failure to confirm. *Am. J. Psychiatry* 133 706–709. 10.1176/ajp.133.6.706 1275104

[B2] Aebi M. Winkler Metzke C. Steinhausen H.-C. (2010). Accuracy of the DSM-oriented attention problem scale of the child behavior checklist in diagnosing attention-deficit hyperactivity disorder.*J*. *Atten. Disord.* 13 454–463. 10.1177/1087054708325739 19372495

[B3] American Psychiatric Association [APA] (2005). *Leitlinien Zur Behandlung Der Borderline Persönlichkeitsstörung*. Bern: Huber.

[B4] American Psychological Association [APA] (1975). Report of the task force on sex bias and sex-role stereotyping in psychotherapeutic practice. *Am. Psychol.* 30 1169–1175. 10.1037/0003-066X.30.12.1169 1200474

[B5] American Psychological Association [APA] (1978). Guidelines for therapy with women. *Psychologist* 33 1122–1123. 10.1037/0003-066X.33.12.1122 736350

[B6] American Psychiatric Association [APA] (2000). *Diagnostic and Statistical Manual of Mental Disorders* 4th Edn. Washington, DC: American Psychiatric Association.

[B7] Angst J. Gamma A. Gastpar M. Lépine J.-P. Mendlewicz J. Tylee A. (2002). Gender differences in depression - epidemiological findings from the european depres I and II Studies. *Eur*. *Arch. Psychiatry Clin. Neurosci.* 252 201–209. 10.1007/s00406-002-0381-6 12451460

[B8] Arvilommi P. Suominen K. S. Mantere O. K. Leppämäki S. Valtonen H. Isometsä E. T. (2007). Adequacy of treatment received by diagnosed and undiagnosed patients with bipolar I and II disorders. *J. Clin. Psychiatry* 68 102–110. 10.4088/JCP.v68n0114 17284137

[B9] Austin P. C. (2011). An introduction to propensity score methods for reducing the effects of confounding in observational studies. *Mul. Behav. Res.* 46 399–424. 10.1080/00273171.2011.568786 PMC3144483 21818162

[B10] Bartak A. Spreeuwenberg M. D. Andrea H. Busschbach J. J. Croon M. A. Verheul R. (2009). The use of propensity score methods in psychotherapy research: a practical application. *Psychother. Psychosom.* 78 26–34. 10.1159/000162298 18852499

[B11] Bertakis K. D. Helms L. J. Callahan E. J. Azari R. Leigh P. Robbinsm J. A. (2001). Patient gender differences in the diagnosis of depression in primary care. *J. Womens Health Gender-Based Med.* 10 689–698. 10.1089/15246090152563579 11571099

[B12] Biederman J. Faraone S. V. Doyle A. Lehman B. K. Kraus I. Perrin J. (1993). Convergence of the child behavior checklist with structured interview-based psychiatric diagnoses of ADHD children with and without comorbidity. *J. Child Psychol. Psychiatry* 34 1241–1251. 10.1111/j.1469-7610.1993.tb01785.x 8245144

[B13] Blashfield R. K. Herkov M. J. (1996). Investigating clinician adherence to diagnosis by criteria: a replication of Morey and Ochoa (1989). *J. Personal. Disord.* 10 219–228. 10.1521/pedi.1996.10.3.219

[B14] Blumenthal-Barby J. S. Krieger H. (2015). Cognitive biases and heuristics in medical decision making: a critical review using a systematic search strategy. *Med*. *Decis. Mak.* 35 539–557. 10.1177/0272989X14547740 25145577

[B15] Bronisch T. Mombour W. (1994). Comparison of a diagnostic checklist with a structured interview for the assessment of DSM-III-R and ICD-10 personality disorders. *Psychopathology* 27 312–320. 10.1159/000284889 7846256

[B16] Broverman I. K. Broverman D. M. Clarkson F. E. Rosenkrantz P. S. Vogel S. R. (1970). Sex-role stereotypes and clinical judgments of mental health. *J*. *Consult. Clin. Psychol.* 34 1–7. 10.1037/h0028797 5436460

[B17] Brownstein A. L. (2003). Biased predecision processing. *Psychol. Bull.* 129 545–568. 10.1037/0033-2909.129.4.545 12848220

[B18] Bruchmüller K. Margraf J. Schneider S. (2012). Is ADHD diagnosed in accord with diagnostic criteria? Overdiagnosis and influence of client gender on diagnosis. *J. Consult. Clin. Psychol.* 80 128–138. 10.1037/a0026582 22201328

[B19] Bruchmüller K. Margraf J. Suppiger A. Schneider S. (2011). Popular or unpopular? Therapists’ use of structured interviews and their estimation of patient acceptance. *Behav. Ther.* 42 634–643. 10.1016/j.beth.2011.02.003 22035992

[B20] Bruchmüller K. Meyer T. D. (2009). Diagnostically irrelevant information can affect the likelihood of a diagnosis of bipolar disorder. *J*. *Affect. Disord.* 116 148–151. 10.1016/j.jad.2008.11.018 19103463

[B21] Case S. M. Hatala R. Blake J. Golden G. S. (1999). Does sex make a difference? Sometimes it does and sometimes it doesn’t. *Acad. Med.* 74 37–40. 10.1097/00001888-199910000-00034 10536588

[B22] Crosby J. P. Sprock J. (2004). Effect of patient sex, clinician sex, and sex role on the diagnosis of antisocial personality disorder: models of underpathologizing and overpathologizing biases. *J. Clin. Psychol.* 60 583–604. 10.1002/jclp.10235 15141394

[B23] Cwik J. C. Teismann T. (2016). Misclassification of self-directed violence. *Clin. Psychol. Psychother.* 10.1002/cpp.2036 [Epub head of print]. 27481725

[B25] D’Agostino R. B. Jr. (1998). Tutorial in biostatistics: propensity score methods for bias reduction in the comparison of a treatment to a non-randomized control group. *Statis. Med.* 17 2265–2281. 10.1002/(SICI)1097-0258(19981015)17:19<2265::AID-SIM918>3.0.CO;2-B 9802183

[B26] Drake R. E. Alterman A. I. Rosenberg S. R. (1993). Detection of substance use disorders in severely mentally ill patients. *Commun. Ment*. *Health J.* 29 175–192. 10.1007/BF00756343 8500289

[B27] Ehlert U. (2007). Eine psychotherapie ist immer nur so gut wie ihre diagnostik. *Verhaltenstherapie* 17 81–82. 10.1159/000103156

[B28] Flanagan E. H. Blashfield R. K. (2005). Gender acts as a context for interpreting diagnostic criteria. *J*. *Clin. Psychol.* 61 1485–1498. 10.1002/jclp.20202 16173082

[B29] Ford M. R. Widiger T. A. (1989). Sex bias in the diagnosis of histrionic and antisocial personality disorders. *J*. *Consult. Clin. Psychol.* 57 301–305. 10.1037/0022-006X.57.2.301 2708619

[B30] Garb H. N. (1996). The Representativeness and past-behavior heuristics in clinical judgment. *Prof. Psychol. Res. Pract.* 27 272–277. 10.1037/0735-7028.27.3.272

[B31] Garb H. N. (1997). Race bias, social class bias, and gender bias in clinical judgment. *Clin. Psychol. Sci. Pract.* 4 99–120. 10.1111/j.1468-2850.1997.tb00104.x

[B32] Garb H. N. (1998). *Studying the Clinician: Judgment Research and Psychological Assessment*. Washington, DC: American Psychological Association.

[B33] Garb H. N. (2007). Computer-administered interviews and rating scales. *Psychol. Assessm.* 19 4–13. 10.1037/1040-3590.19.1.4 17371119

[B34] Garb H. N. (2013). Cognitive and social factors influencing clinical judgment in psychiatric practice. *World Psychiatry* 12 108–110. 10.1002/wps.20045 23737410 PMC3683253

[B35] Gomes B. Abramowitz S. I. (1976). Sex-related patient and therapist effects on clinical judgment. *Sex Roles* 2 1–13. 10.1007/BF00289293

[B36] Groenier M. Pieters J. M. Hulshof C. D. Wilhelm P. Witteman C. L. M. (2008). Psychologists’ judgements of diagnostic activities: deviations from a theoretical model. *Clin. Psychol. Psychother.* 15 256–265. 10.1002/cpp.587 19115446

[B37] Hansen F. J. Reekie L.-J. (1990). Sex differences in clinical judgment of male and female therapists. *Sex Roles* 23 51–64. 10.1007/BF00289879

[B38] Haynes S. N. Williams A. E. (2003). Case formulation and design of behavioral treatment programs: matching treatment mechanisms to causal variables for behavior problems. *Euro. J. Psychol. Assess.* 19 164–174. 10.1027//1015-5759.19.3.164

[B39] Heatherington L. Stets J. Mazzarella S. (1986). Whither the bias: the female client’s ‘edge’ in psychotherapy? *Psychother. Theor. Res. Pract. Train.* 23 252–256. 10.1037/h0085606

[B40] Hiller W. Zaudig M. Mombour W. Bronisch T. (1993). Routine psychiatric examinations guided by ICD-10 diagnostic checklists (International diagnostic checklists). *Eur*. *Arch. Psychiatry Clin. Neurosci.* 242 218–223. 10.1007/BF02189966 8461348

[B41] IBM Corporation (2013). *SPSS.* Armonk, NY: IBM Corporation.

[B42] In-Albon T. Suppiger A. Schlup B. Wendler S. Margraf J. Schneider S. (2008). Validität des diagnostischen interviews bei psychischen störungen (DIPS Für DSM-IV-TR). *Z. Klin. Psychol. Psychother.* 37 33–42. 10.1026/1616-3443.37.1.33

[B43] Joiner T. E. Jr. Walker R. L. Pettit J. W. Perez M. Cukrowicz K. C. (2005). Evidence-based assessment of depression in adults. *Psychol. Assess.* 17 267–277. 10.1037/1040-3590.17.3.267 16262453

[B44] Kales H. C. Neighbors H. W. Blow F. C. Taylor K. K. K. Gillon L. Welsh D. E. (2005). Race, gender, and psychiatrists’ diagnosis and treatment of major depression among elderly patients. *Psychiatr. Ser.* 56 721–728. 10.1176/appi.ps.56.6.721 15939950

[B45] Langer E. J. Abelson R. P. (1974). A patient by any other name: clinician group difference in labeling bias. *J. Consul. Clin. Psychol.* 42 4–9. 10.1037/h0036054 4814097

[B46] Lewis R. Lamdan R. M. Wald D. Curtis M. (2006). Gender bias in the diagnosis of a geriatric standardized patient: a potential confounding variable. *Acad*. *Psychiatry* 30 392–396. 10.1176/appi.ap.30.5.392 17021147

[B47] López S. R. Smith A. Wolkenstein B. H. Charlin V. (1993). Gender bias in clinical judgment: an assessment of the analogue method’s transparency and social desirability. *Sex Roles* 28 35–45. 10.1007/BF00289746

[B48] Margraf J. Schneider S. (2009). “Diagnostik psychischer störungen mit strukturierten interviews,” in *Lehrbuch Der Verhaltenstherapie, Band 1: Grundlagen, Diagnostik, Verfahren, Rahmenbedingungen* 3rd Edn eds Margraf J. Schneider S. (Heidelberg: Springer) 344–345.

[B49] Meyer F. Meyer T. D. (2009). The misdiagnosis of bipolar disorder as a psychotic disorder: some of its causes and their influence on therapy. *J*. *Affect. Disord.* 112 174–183. 10.1016/j.jad.2008.04.022 18555536

[B50] Mokros A. Hollerbach P. Nitschke J. Eher R. Habermeyer E. (2013). Normative data for the psychopathy checklist-revised in german-speaking countries - a meta-analysis. *Crim. Just. Behav.* 40 1397–1412. 10.1177/0093854813492519

[B51] Möller-Leimkühler A. M. (2005). Geschlechterrolle und psychische erkrankung. *J. Neurol. Neurochir. Psychiatr.* 6 29–35.

[B52] Morey L. C. Ochoa E. S. (1989). An investigation of adherence to diagnostic criteria: clinical diagnosis of the DSM-III personality disorders. *J*. *Personal. Disord.* 3 180–192. 10.1521/pedi.1989.3.3.180

[B53] Morschitzky H. (2009). *Angststörungen: Diagnostik, Konzepte, Therapie, Selbsthilfe* 4th Edn. Wien: Springer.

[B54] North C. S. Pollio D. E. Thompson S. J. Ricci D. A. Smith E. M. Spitznagel E. L. (1997). A comparison of clinical and structured interview diagnoses in a homeless mental health clinic. *Commun. Mental Health J.* 33 531–543. 10.1023/A:1025052720325 9435999

[B55] Ramirez Basco M. Bostic J. Q. Davies D. Rush A. J. Witte B. Hendrickse W. (2000). Methods to improve diagnostics accuracy in a community mental health setting. *Am. J. Psychiatry* 157 1599–1605. 10.1176/appi.ajp.157.10.1599 11007713

[B56] Rosenbaum P. R. Rubin D. B. (1983). The central role of the propensity score in observational studies for causal effects. *Biometrika* 70 41–55. 10.1093/biomet/70.1.41

[B57] Rosenhan D. L. Seligman M. E. P. (1989). *Abnormal Psychology* 2nd Edn. New York, NY: Norton.

[B58] Schmidt N. B. Salas D. Bernert R. Schatschneider C. (2005). Diagnosing agoraphobia in the context of panic disorder: examining the effect of dsm-iv criteria on diagnostic decision-making. *Behav*. *Res. Ther.* 43 1219–1229. 10.1016/j.brat.2004.09.004 16005707

[B59] Schneider S. Döpfner M. (2004). Leitlinien zur diagnostik und psychotherapie von angst- und phobischen störungen im kindes- und jugendalter: ein evidenzbasierter diskussionsvorschlag. *Kind. Entwickl.* 13 80–96. 10.1026/0942-5403.13.2.80

[B60] Seedat S. Scott K. M. Angermeyer M. C. Berglund P. Bromet E. J. Brugha T. S. (2009). Cross-national associations between gender and mental disorders in the WHO world mental health surveys. *Arch*. *Gen. Psychiatry* 66 785–795. 10.1001/archgenpsychiatry.2009.36 PMC2810067 19581570

[B61] Silverman W. K. Ollendick T. H. (2005). Evidence-based assessment of anxiety and its disorders in children and adolescents. *J*. *Clin. Child Adolesc. Psychol.* 34 380–411. 10.1207/s15374424jccp3403_2 16026211

[B62] Spitzer R. L. Gibbon M. Skodol A. E. Williams J. B. First M. B. (1991). “Falldarstellungen diagnostisches und statistisches manual psychischer störungen (DSM-III-R),” in *Falldarstellungen Diagnostisches Und Statistisches Manual Psychischer Störungen (DSM-III-R)* eds Spitzer R. L. Gibbon M. Skodol A. E. Williams J. B. First M. B. (Weinheim: Beltz Verlag) 257–258.

[B63] Sprock J. (1996). Abnormality ratings of DSM-III-R personality disorder criteria for males vs. *females*. *J. Nervous Ment. Dis.* 184 314–316. 10.1097/00005053-199605000-00008 8627278

[B64] Suppiger A. In-Albon T. Hendriksen S. Hermann E. Margraf J. Schneider S. (2009). Acceptance of structured diagnostic interviews for mental disorders in clinical practice and research settings. *Behav. Ther.* 40 272–279. 10.1016/j.beth.2008.07.002 19647528

[B65] Suppiger A. In-Albon T. Herren C. Bader K. Schneider S. Margraf J. (2008). Reliabilität des diagnostischen interviews bei psychischen störungen (DIPS Für DSM-IV-TR) unter klinischen routinebedingungen. *Verhaltenstherapie* 18 237–244. 10.1159/000169699

[B66] Teri L. (1982). Effects of sex and sex-role style on clinical judgment. *Sex Roles* 8 639–649. 10.1007/BF00289897

[B67] Tversky A. Kahneman D. (1974). Judgment under uncertainty: heuristics and biases. *Science* 185 1124–1131. 10.1126/science.185.4157.1124 17835457

[B68] Vaughn A. J. Hoza B. (2013). The incremental utility of behavioral rating scales and a structured diagnostic interview in the assessment of attention-deficit/hyperactivity disorder. *J. Emot. Behav. Disord.* 21 227–239. 10.1177/1063426611427910

[B69] Vicente B. Kohn R. Levav I. Espejo F. Saldivia S. Sartorius N. (2007). Training primary care physicians in chile in the diagnosis and treatment of depression. *J. Affect. Disord.* 98 121–127. 10.1016/j.jad.2006.07.006 16928402

[B70] Vredenburg K. Krames L. Flett G. L. (1986). Sex differences in the clinical expression of depression. *Sex Roles* 14 37–49. 10.1007/BF00287846

[B71] Weinberger A. H. Maciejewski P. K. McKee S. A. Reutenauer E. L. Mazure C. M. (2009). Gender differences in associations between lifetime alcohol, depression, panic disorder, and posttraumatic stress disorder and tobacco withdrawal. *Am. J. Addict.* 18 140–147. 10.1080/10550490802544888 19283566 PMC2874459

[B72] Westen D. Shedler J. (2000). A prototype matching approach to diagnosing personality disorders: toward DSM-V. *J*. *Personal. Disord.* 14 109–126. 10.1521/pedi.2000.14.2.109 10897462

[B73] World Health Organization [WHO] (1992). *The ICD-10 Classification of Mental and Behavioural Disorders: Clinical Descriptions and Diagnostic Guidelines.* Geneva: World Health Organization.

[B74] Widiger T. A. Spitzer R. L. (1991). Sex bias in the diagnosis of personality disorders: conceptual and methodological issues. *Clin*. *Psychol. Rev.* 11 1–22.

[B75] Wittchen H.-U. Jacobi F. (2005). Size and burden of mental disorders in europe: a critical review and appraisal of 27 studies. *Eur*. *Neuropsychopharmacol.* 15 357–376. 10.1016/j.euroneuro.2005.04.012 15961293

[B76] Wittchen H.-U. Unland H. (1991). Neue ansätze zur symptomerfassung und diagnosestellung nach ICD-10 Und DSM-III-R: strukturierte und standardisierte interviews. *Z. Klin. Psychol.* 20 321–342.

[B77] Witteman C. Kunst H. (1997). Planning the treatment of a depressed patient. *Clin. Psychol. Psychother.* 4 157–171. 10.1002/(SICI)1099-0879(199709)4:3<157::AID-CPP127>3.3.CO;2-#

[B78] Wolkenstein L. Bruchmüller K. Schmid P. Meyer T. D. (2011). Misdiagnosing bipolar disorder - do clinicians show heuristic biases? *J. Affect. Disord.* 130 405–412. 10.1016/j.jad.2010.10.036 21093059

[B79] Wrobel N. H. (1993). Effect of patient age and gender on clinical decisions. *Profess. Psychol. Res. Pract.* 24 206–212. 10.1037/0735-7028.24.2.206

[B80] Zaudig M. Wittchen H.-U. Sass H. (2000). *DSM-IV Und ICD-10 Fallbuch: Fallübungen Zur Differentialdiagnose Nach DSM-IV Und ICD-10*. Göttingen: Hogrefe.

[B81] Zimmerman M. (2016). A review of 20 years of research on overdiagnosis and underdiagnosis in the rhode island methods to improve diagnostic assessment and services (MIDAS) project. *Can. J. Psychiatry* 61 71–79. 10.1177/0706743715625935 27253697 PMC4784239

[B82] Zimmerman M. Jampala V. C. Sierles F. S. Taylor M. A. (1993). DSM-III and DSM-III-R: what are american psychiatrists using and why? *Comprehen. Psychiatry* 34 360–374. 10.1016/0010-440X(93)90060-H 8131380

[B83] Zimmerman M. Mattia J. I. (1999). Psychiatric diagnosis in clinical practice: is comorbidity being missed? *Comprehen. Psychiatry* 40 182–191. 10.1016/S0010-440X(99)90001-9 10360612

